# Dual-Ascent-Inspired Transformer for Compressed Sensing

**DOI:** 10.3390/s25072157

**Published:** 2025-03-28

**Authors:** Rui Lin, Yue Shen, Yu Chen

**Affiliations:** SCS Laboratory, Department of Human and Engineered Environmental Studies, Graduate School of Frontier Sciences, The University of Tokyo, 5-1-5, Kashiwa-no-ha, Kashiwa City 277-8563, Chiba, Japan; lin-rui398@g.ecc.u-tokyo.ac.jp (R.L.); shenyue@s.h.k.u-tokyo.ac.jp (Y.S.)

**Keywords:** compressed sensing, image reconstruction, deep unfolding network, dual ascent, transformer

## Abstract

Deep learning has revolutionized image compressed sensing (CS) by enabling lightweight models that achieve high-quality reconstruction with low latency. However, most deep neural network-based CS models are pre-trained for specific compression ratios (CS ratios), limiting their flexibility compared to traditional iterative algorithms. To address this limitation, we propose the Dual-Ascent-Inspired Transformer (DAT), a novel architecture that maintains stable performance across different compression ratios with minimal training costs. DAT’s design incorporates the mathematical properties of the dual ascent method (DAM), leading to accelerated training convergence. The architecture features an innovative asymmetric primal–dual space at each iteration layer, enabling dimension-specific operations that balance reconstruction quality with computational efficiency. We also optimize the Cross Attention module through parameter sharing, effectively reducing its training complexity. Experimental results demonstrate DAT’s superior performance in two key aspects: First, during early-stage training (within 10 epochs), DAT consistently outperforms existing methods across multiple CS ratios (10%, 30%, and 50%). Notably, DAT achieves comparable PSNR to the ISTA-Net+ baseline within just one epoch, while competing methods require significantly more training time. Second, DAT exhibits enhanced robustness to variations in initial learning rates, as evidenced by loss function analysis during training.

## 1. Introduction

Compressed sensing (CS) is an important technique in signal processing that integrates a specialized sampling process with a reconstruction process [1]. Implementing CS involves designing algorithms or models for both the sampling and reconstruction stages. During sampling, the signal undergoes a linear random transformation, which simultaneously compresses it. During reconstruction, the original signal can be reconstructed from measurements that are significantly fewer than those required by the Nyquist sampling rate [2]. CS theory demonstrates that, if a signal exhibits sparsity in a certain transform domain, it can be reconstructed with high probability [3]. Hence, CS enables a reduction in both sampling rate and storage requirements while maintaining high speed signal acquisition, transmission, and reconstruction. This novel sampling strategy is hardware-friendly, making CS technology particularly successful in various imaging applications, such as medical imaging [4], single-pixel cameras [5], wireless remote monitoring, and snapshot compressive imaging [6].

Mathematically, the aim of reconstruction is to infer the original signal $x\in R^{N}$ from its random measurements $y\in R^{M}$. The sampling process of CS can be expressed as

(1) y=Ax,

where $A\in R^{M\times N}$ is a linear random projection matrix, and the compressed sensing ratio is defined as $\frac{M}{N}$. Since M≪N, this inverse problem is typically ill-posed. To achieve a reliable reconstruction, traditional algorithms usually are boiled down to solving an optimization problem:

(2) $$\arg\min_{x}\frac{1}{2}{∥Ax-y∥}_{2}^{2}+\lambda R\left(x\right),$$

where $\frac{1}{2}{∥Ax-y∥}_{2}^{2}$ denotes the data fidelity term, $\lambda R\left(x\right)$ denotes the prior term with a regularization parameter λ. Specifically, when the regularization function $R\left(x\right)$ is chosen to be ${∥Bx∥}_{1}$, with $B\in R^{p\times n}$ being a sparsifying operator, e.g., Wavelet Transform [7], this optimization is known as the LASSO problem [8].

Most traditional CS methods exploit structural sparsity as prior knowledge and solve a sparsity-regularized optimization problem iteratively [2]. The prior term typically involves a predefined operator *B* that transforms images into a domain where they exhibit sparsity, such as the discrete cosine transform (DCT) or wavelet transform. These methods offer strong theoretical guarantees and stable convergence properties in most cases. However, they suffer from high computational complexity and difficulties in selecting the optimal transform and tuning hyperparameters for effective reconstruction.

With the advent of deep learning, several deep network-based CS models have been proposed [9,10]. Though these models can rapidly infer solutions to CS problems, most of them rely on black-box architectures that do not fully incorporate the theoretical advantages of traditional optimization-based methods [11].

Recently, deep unfolding networks (DUNs) have gained prominence due to their improved interpretability. These models integrate optimization algorithms into neural network architectures by unfolding iterative processes, providing a structured approach to solving CS problems [11]. However, some high-performance DUNs require an extensive number of parameters. For instance, PRL-RND+ [12] has 55 M parameters, and Idm [13] has 100 M parameters, leading to increased computational costs and training challenges. Although lightweight models [14,15,16] with 0.8–2.4 M parameters have been introduced to mitigate memory usage and inference latency, training efficiency remains a largely overlooked issue. Unlike classical optimization-based approaches, which allow for flexible adaptation to different compression ratios, most DUNs require pre-trained models tailored to specific CS ratios. For individual users in practice, there is often a need to balance storage efficiency and reconstruction quality, which necessitates a DUN that can achieve fast convergence, low training complexity, and high reconstruction performance to facilitate personalized CS applications on personal computing devices.

To address these challenges, this paper proposes an efficient DUN module inspired by the classical dual-ascent method (DAM), termed the Dual-Ascent-Inspired Transformer (DAT). This module serves as the fundamental iterative layer in a lightweight image CS workflow. The complete DAT-based workflow is an end-to-end pipeline, processing raw images as inputs and producing reconstructed images as outputs, encompassing both the sampling and reconstruction stages. Within the deep reconstruction module, compressed measurements pass through multiple iterative layers composed of DAT modules to recover the original image. Each DAT module consists of three key submodules: Cross Attention (CA), High-pass Filter (HPF), and Dual Ascent (DA). By leveraging the mathematical properties of the DAM, DAT requires significantly less training data, hence enabling the high-quality reconstruction with fewer parameters.

## 2. Related Work

### 2.1. Deep Unfolding Networks

In recent years, neural network architectures have been increasingly applied to compressed sensing (CS), drawing inspiration from their success in image classification tasks. Early works, such as CNN-based models, learned the end-to-end mapping of compressed measurements to reconstructed images [9]. However, purely data-driven models suffer from interpretability issues due to their black-box nature, which limits their ability to integrate theoretical insights for further performance improvements. This limitation arises because these models rely on stacking convolutional and filtering layers to optimize a nonlinear transformation that maps input images to output images. While this approach can be effective, it disregards the fact that certain image-processing tasks, such as compressive sensing, have well-established mathematical interpretations for their intermediate steps. Black-box models offer limited control over these intermediate processes, making it difficult to verify whether the representations learned during training align with the theoretical priors inherent to the given image-processing task.

To address these limitations, a new class of models, known as deep unfolding networks (DUNs), are invented by integrating convolutional layers with traditional optimization-based algorithms. The core idea behind DUNs is to replace each iteration step of a conventional optimization algorithm with a trainable module. For instance, Zhang et al. proposed ISTA-Net [11], which incorporates convolutional layers into the iterative shrinkage-thresholding algorithm (ISTA) [17] for image reconstruction. A similar strategy was adopted in ADMM-CSNet [18], which was inspired by the alternating direction method of multipliers (ADMM) [19]. While these models employ a learning-based approach in the reconstruction process, their sampling modules still rely on handcrafted sensing matrices, potentially limiting their performance. To address this issue, Zhang et al. proposed ISTA-Net++ [20], which replaces the handcrafted sensing matrix with trainable convolutional layers, enabling more effective feature propagation across blocks and significantly improving reconstruction quality. Further advancements include FSOINET [21], which suggests executing soft-thresholding functions in a high-dimensional feature space rather than the original pixel space to enhance image reconstruction quality.

The studies reviewed above show that DUNs have significantly improved CS reconstruction quality from various perspectives. Next, we shall focus on the bilevel optimization formulation that underpins these methods.

DUNs are commonly formulated as solving a bilevel optimization problem:

(3) $$\begin{array}{cc} \; & \min_{\theta }\sum_{i=1}\ell_{\theta }\left(x_{i}^{*},x_{i}\right),\\ \; & s.t.\;x_{i}^{*}=\arg\min_{x}\frac{1}{2}{∥Ax-y_{i}∥}_{2}^{2}+\lambda R\left(x\right), \end{array}$$

where θ denotes the trainable parameters, and $\ell_{\theta }\left(x_{i}^{*},x_{i}\right)$ denotes the loss function measuring the difference between the original image $x_{i}$ and the reconstructed image $x_{i}^{*}$.

DUN-based approaches often integrate efficient CNN-based filtering operations into the optimization methods, including the proximal gradient descent (PGD) algorithm [11], approximate message passing (AMP) [22], the inertial proximal algorithm for non-convex optimization (iPiano) [23], and ADMM-based methods [19]. Each of these optimization algorithms lead to a distinct optimization-inspired DUN architecture.

Although DUNs outperform conventional iterative methods in both reconstruction quality and inference speed, their computational efficiency during training is often overlooked. Most existing DUN designs focus on enhancing the richness of extracted image features while neglecting the convergence speed of the underlying optimization process. Consequently, some recent DUNs, when trained on limited datasets, underperform compared with earlier models such as ISTA-Net.

To address this issue, this paper proposes a streamlined and efficient DUN architecture that leverages the fast convergence properties of the classical dual-ascent method, in order to accelerate training while minimizing memory usage and preserving high reconstruction accuracy.

### 2.2. Vision Transformer

The original Transformer [24] was designed for natural language processing (NLP). Inspired by its success, the Vision Transformer (ViT) [25] was introduced to process images by segmenting them into flat 16 × 16 patches, treating each patch as a token. This approach successfully extended the Transformer architecture to image-classification tasks. Since then, Transformers have emerged as a new paradigm in deep learning, alongside CNNs.

In recent years, Transformer-based deep unfolding networks have gained popularity in compressed sensing. For example, CSformer [16], proposed by Ye et al., integrates Transformers and CNNs to fuse intermediate features in a dual-path and black-box manner, achieving significant improvements in reconstruction quality over previous methods. Similarly, TransCS [15], proposed by Shen et al., interprets the network as an iterative equivalent of ISTA, incorporating gradient descent and soft-thresholding iterations. In this framework, the Transformer serves as an encoder, mapping data from the original space to a sparse feature space, thereby enhancing interpretability. Building on this idea, Song et al. introduced Octuf [14], which employs two types of asymmetric cross-attention modules: one leveraging inertial information in the feature space and the other capturing cross-information between the original and feature spaces. This design significantly improves both reconstruction speed and quality while reducing parameter overhead.

These advancements highlight the remarkable potential of Transformers in compressed sensing, particularly in enhancing reconstruction quality and efficiency. In this paper, we further extend this direction by leveraging Transformers not only to integrate inertial information from the dual feature space into variables in the original space but also to introduce residual information from the measurement space into the dual space at each iterative layer. This integration, achieved through the dual-ascent mechanism, can significantly accelerate the convergence of the training process.

### 2.3. Dual-Ascent Method

The dual-ascent method encompasses a family of optimization algorithms that facilitate accelerated convergence, global optimality, and distributed computing. This family includes the classical Dual Ascent, the Method of Multipliers, and the Alternating Direction Method of Multipliers (ADMM) [19]. Among these algorithms, ADMM has been widely applied to the LASSO problem [26] in unconstrained optimization:

(4) $$\begin{array}{c} \min_{x}f\left(x\right)+g\left(x\right), \end{array}$$

which can be reformulated as a constrained optimization problem if there exists a variable *z* such that Ax+Bz=c:

(5) $$\begin{array}{cc} \; & \min_{x,z}f\left(x\right)+g^{\prime }\left(z\right),\\ \; & s.t.\;Ax+Bz=c, \end{array}$$

where $A\in R^{p\times n}$, $B\in R^{p\times m}$, $x\in R^{n}$, $z\in R^{m}$, and $c\in R^{p}$. Following the method of multipliers, we define the augmented Lagrangian as

(6) $$\begin{array}{c} L\left(x,z,v\right)=f\left(x\right)+g^{\prime }\left(z\right)+v^{T}\left(Ax+Bz-c\right)+\frac{1}{2}\rho {∥Ax+Bz-c∥}_{2}^{2}, \end{array}$$

where $v\in R^{p}$ denotes dual variable and ρ>0. Given the initial variables $\left(x_{0},z_{0},v_{0}\right)$, for problem (5), the ADMM algorithm includes the following steps:

(7) $$\begin{array}{c} x_{k+1}=\arg\min_{x}L\left(x,z_{k},v_{k}\right), \end{array}$$

(8) $$\begin{array}{c} \begin{array}{c} \;z_{k+1}=\arg\min_{z}L\left(x_{k+1},z,v_{k}\right), \end{array} \end{array}$$

(9) $$\begin{array}{c} \begin{array}{c} \;\;v_{k+1}=v_{k}+\rho \left(Ax_{k+1}-Bz_{k+1}-c\right). \end{array} \end{array}$$

Here, Equation (9) represents the dual-ascent step. When ADMM is applied to compressed sensing for image reconstruction—specifically, solving the LASSO problem (2)—the classical algorithm follows:

(10) $$\begin{array}{c} \;\;\;\;\;\;\;\;x_{k+1}={\left(A^{T}A+\rho B^{T}B\right)}^{-1}\left(A^{T}y+\rho B^{T}\left(z_{k}-v_{k}\right)\right), \end{array}$$

(11) $$\begin{array}{c} \begin{array}{c} z_{k+1}=S_{\lambda /\rho }\left(Bx_{k+1}+v_{k}\right), \end{array} \end{array}$$

(12) $$\begin{array}{c} \begin{array}{c} v_{k+1}=v_{k}+Bx_{k+1}-z_{k+1}, \end{array} \end{array}$$

where $S_{\lambda /\rho }\left(•\right)$ denotes a Soft-Thresholding function [27] with the parameters λ,ρ, which is defined as

(13) $$S_{\lambda /\rho }\left(x\right)=\left\{\begin{array}{cc} x+\lambda /\rho & x\leq -\lambda /\rho ,\\ 0 & \left|x\right|<\lambda /\rho ,\\ x-\lambda /\rho & x\geq \lambda /\rho . \end{array}\right.$$

Notably, the first step of calculating Equation (10) involves matrix inversion related to the sensing matrix *A*, which has a computational complexity of approximately $O(N^{3})$. As image size increases, the dimensions of the sensing matrix grow accordingly, leading to substantial computational overhead due to the inversion operation.

To address this, we employ the approximate ADMM algorithm [28] introduced in our previous work. As will be detailed in Section 3.1, this approach retains the dual-ascent step, Equation (9)—where dual variables are updated by incorporating residual information from the measurement space—while eliminating the costly matrix inversion, thereby ensuring lower computational complexity. This serves as a strong mathematical foundation for the potential acceleration of deep unfolding networks (DUNs) based on this optimization strategy.

## 3. Proposed Method

In this section, we first propose an inertial-dual-ascent form of the Approximate Alternating Direction Method of Multipliers (AADMM), based on our previous work [28], which is the algorithmic foundation of the reconstruction module in our deep unfolding network model. Next, we will introduce the framework of DAT, which includes the sampling module and the reconstruction module. For the reconstruction module, we will separately discuss the specific implementation processes of the Cross Attention (CA) submodule, High-pass Filter (HPF) submodule, and Dual Ascent (DA) submodule, as well as other auxiliary modules.

### 3.1. Inertial-Dual-Ascent Form of AADMM

In our previous work [28], we proposed the Approximate Alternating Direction Method of Multipliers (AADMM), a novel approach to applying the ADMM algorithm to the LASSO problem by reformulating the original problem (2) as follows (for the case where the number of inner loop iterations J=1 in the original formulation):

(14) $$\begin{array}{cc} \; & \arg\min_{x,z}\frac{1}{2}{∥z-y∥}_{2}^{2}+\lambda {∥w∥}_{1},\\ \; & s.t.\;A^{F}w=z, \end{array}$$

where $w=Fx\in R^{D}$, $A^{F}=AF^{-1}\in R^{M\times D}$, and $F\in R^{D\times N}$ denotes a sparse mapping operator. Given the initial variables $\left(w_{0},z_{0},v_{0}\right)$, the iterative updates are defined as follows:

(15) $$\begin{array}{cc} s_{k+1} & =w_{k}-\gamma {\left(A^{F}\right)}^{T}\left(A^{F}w_{k}-z_{k}+\frac{1}{\rho }v_{k}\right), \end{array}$$

(16) $$\begin{array}{c} \begin{array}{cc} w_{k+1} & =S_{\gamma \lambda /\rho }\left(s_{k+1}\right), \end{array} \end{array}$$

(17) $$\begin{array}{c} \begin{array}{cc} z_{k+1} & =\frac{1}{1+\rho }\left(y+\rho \left(A^{F}w_{k+1}+\frac{1}{\rho }v_{k}\right)\right), \end{array} \end{array}$$

(18) $$\begin{array}{c} \begin{array}{cc} v_{k+1} & =v_{k}+\rho \left(A^{F}w_{k+1}-z_{k+1}\right), \end{array} \end{array}$$

$$\begin{array}{c} \begin{array}{c} \cdots \end{array} \end{array}$$

(19) $$\begin{array}{c} \begin{array}{cc} x^{*} & =F^{-1}w_{K}, \end{array} \end{array}$$

where *K* denotes the final iteration, γ>0 and ρ>0 are step-size-related parameters, and $S_{\gamma \lambda /\rho }\left(•\right)$ represents a nonlinear filter function [27] parameterized by γ,λ, and ρ. The variables $x_{k}\in R^{N}$ and $z_{k}\in R^{M}$ serve as the solutions to the two subproblems at the *k*-th iteration, while $s_{k}\in R^{N}$ is an intermediate variable obtained through gradient descent, and $v_{k}\in R^{M}$ is the dual variable.

Unlike the conventional ADMM formulation in Equation (10), this method eliminates the need for matrix inversion involving the sensing matrix *A*. Notably, matrix *B* in ADMM often has a known or easily computable inverse, such as in total variation (TV) regularization [29]. As a result, the AADMM approach significantly reduces computational complexity, reinforcing the feasibility of constructing a deep unfolding network based on this algorithm while preserving its fast convergence characteristics.

In this study, we further refine the AADMM formulation by substituting Equation (17) from the (k−1)-th and *k*-th iterations into Equations (15) and (18) at iteration *k*. After rearranging the terms, we derive the following iterative update scheme:

(20) $$\begin{array}{cc} s_{k+1} & =w_{k}-\frac{\gamma }{1+\rho }{\left(A^{F}\right)}^{T}\left(A^{F}w_{k}-y\right)+{\left(A^{F}\right)}^{T}\left(\frac{1}{\rho }v_{k}-\frac{1}{1+\rho }v_{k-1}\right), \end{array}$$

(21) $$\begin{array}{cc} w_{k+1} & =S_{\gamma \lambda /\rho }\left(s_{k+1}\right), \end{array}$$

(22) $$\begin{array}{cc} v_{k+1} & =\frac{1}{1+\rho }v_{k}+\frac{\rho }{1+\rho }\left(A^{F}w_{k+1}-y\right), \end{array}$$

$$\begin{array}{c} \begin{array}{c} \cdots \end{array} \end{array}$$

(23) $$\begin{array}{c} \begin{array}{cc} x^{*} & =F^{-1}w_{K}. \end{array} \end{array}$$

We refer to this reformulated approach as the inertial-dual-ascent form of AADMM. In this formulation, Equation (20) represents a gradient descent step with an inertial term. Prior research [30] has demonstrated that incorporating inertial terms helps mitigate the tendency of gradient descent to become trapped in local minima, thereby improving stability and convergence efficiency. Meanwhile, Equation (22) constitutes a dual-ascent step, a critical feature of ADMM-based methods.

It is important to emphasize that, unlike some previous studies [14] where dual variables were manually separated, our dual variable update step naturally emerges from the Approximate ADMM framework. This ensures that the accelerated convergence properties inherent to ADMM are preserved. Consequently, the deep unfolding network constructed based on this algorithm benefits from a robust mathematical foundation, allowing for significantly enhanced convergence speed and efficiency.

### 3.2. Dual-Ascent-Inspired Transformer

#### 3.2.1. Overall Architecture

The DAT workflow is an end-to-end process designed to reconstruct an image from its compressed measurements. It consists of two main components: a sampling (compression) module and a reconstruction module.

**Figure 1:**
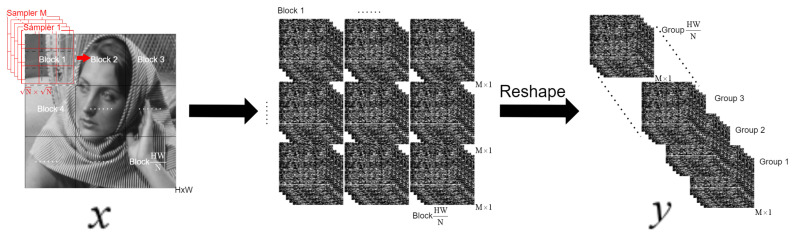
The architecture of the Cross-Block Strategy (CBS) [20] sampling module. The original H×W dimensional tensor *x* is compressed into $M\times \frac{HW}{N}$ dimensional measurement tensor *y* through the sampling module.

**Figure 2:**
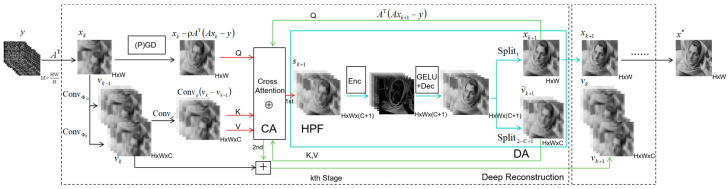
The architecture of deep reconstruction module of DAT. The inputs and outputs of CA (Cross Attention), HPF (High-pass Filter), and DA (Dual Ascent) are indicated by red, blue, and green arrows, respectively. “+” denotes addition, and ⊕ represents the binary operation in the CA module.

In the sampling module, as illustrated in Figure 1, we employ the CBS sampling strategy. Specifically, compression is achieved by applying block-wise convolution on the original image $x\in R^{H\times W}$, followed by reshaping it into an $M\times \frac{HW}{N}$ dimensional measurement tensor *y*. A more detailed explanation will be provided in the subsequent sections. As shown in Figure 2, these measurements are then processed by the initial reconstruction module to obtain the primal variable $x_{0}\in R^{H\times W}$ and the dual variable $v_{-1},v_{0}\in R^{H\times W\times C}$ during the first stage. These initial estimates serve as inputs to the deep reconstruction module, which consists of k layers of DAT iteration. The iterative process progressively refines the reconstruction, ultimately producing the final output image.

In the deep reconstruction module, we use a deep neural network inspired by the inertial-dual-ascent form of AADMM to establish each iteration layer, which can be formulated as (k∈1,2,⋯,K)

(24) $$\begin{array}{ccc} & s_{k+1} & =\left(x_{k}-\rho A^{T}\left(Ax_{k}-y\right)\right)⊕{\mathrm{Conv}}_{e}\left(v_{k}-v_{k-1}\right), \end{array}$$

(25) $$\begin{array}{c} \begin{array}{cc} x_{k+1} & ={\mathrm{Split}}_{1}\left(\mathrm{HPF}\left(s_{k+1}\right)\right), \end{array} \end{array}$$

(26) $$\begin{array}{cc} & \begin{array}{cc} {\tilde{v}}_{k+1} & ={\mathrm{Split}}_{2∼C+1}\left(\mathrm{HPF}\left(s_{k+1}\right)\right), \end{array} \end{array}$$

(27) $$\begin{array}{cc} & \begin{array}{cc} v_{k+1} & =v_{k}+\left[•⊕{\tilde{v}}_{k+1}\right]\left(A^{T}\left(Ax_{k+1}-y\right)\right)\\ \; & =v_{k}+{\mathrm{Split}}_{2∼C+1}\left(\left(A^{T}\left(Ax_{k+1}-y\right)\right)⊕{\tilde{v}}_{k+1}\right), \end{array} \end{array}$$

where $x_{k}\in R^{H\times W}$ is the primal variable, $s_{k}\in R^{H\times W}$ is the intermediate variable resulting from the Cross Attention module, $v_{k}\in R^{H\times W\times C}$ is the dual variable, and ${\tilde{v}}_{k}$ denotes the parameters which are reused. The notation •⊕★ represents a binary operation involving two asymmetric inputs, specifically within the Cross Attention module. The operators ${\mathrm{Conv}}_{e}\left(•\right)$, $\mathrm{HPF}\left(•\right)$, and $\left[•⊕{\tilde{v}}_{k+1}\right]$ correspond to neural network layers with learnable parameters. Furthermore, ${\mathrm{Split}}_{1}\left(•\right)$ and ${\mathrm{Split}}_{2∼C+1}\left(•\right)$ are defined as segmentation operators applied along the C+1 dimension of an H×W×(C+1) tensor. These operators extract the first dimension and the second (C+1)th dimension, respectively, forming new tensors. As illustrated in Figure 2, these components collectively contribute to the deep reconstruction module of DAT.

In DAT, we replace the manually designed parameters in the inertial-dual-ascent form of AADMM with neural network operators containing learnable parameters. This transformation can be illustrated in the following aspects:

1. Compared to Equation (20), Equation (24) replaces the fixed coefficient with a learnable convolutional layer, which encodes the residual of the dual variables between adjacent iteration layers. Additionally, it replaces the linear addition operation with a Cross Attention module, which integrates the result of the gradient descent operator with the residual of the dual variables. This design more effectively incorporates inertial information from the dual space into the primal variable updates, thereby facilitating accelerated convergence while ensuring global stability;
2. Equation (25) represents a proximal mapping step, which essentially functions as a high-pass filter in the sparse space. Following widely adopted practices in Deep Unfolding Networks (DUNs) [14,15,20], we replace the fixed matrix mappings in Equation (25) with convolutional layers containing learnable parameters, allowing for a more accurate transformation between the intermediate variable space and the sparse domain. Additionally, we employ the GELU activation function [31], which shares a similar role with the soft-thresholding function, to perform filtering. This process is encapsulated in the High-pass Filter (HPF) module;
3. In Equations (26) and (27), we introduce ${\tilde{v}}_{k+1}$ as a fixed input to the right side of the Cross Attention module, forming a unified encoder $\left[•⊕{\tilde{v}}_{k+1}\right]$ that integrates the residual term $Ax_{k+1}-y$ into the dual variable update through the Dual Ascent (DA) module. Notably, this encoder not only ensures dimension consistency between the primal and dual variables but also reuses the parameters of the Cross Attention module, so as to avoid employing two independent CA modules. This design can reduce computational complexity and memory consumption, while also providing superior encoding performance compared to the fixed-coefficient approach used in Equation (22).

**Figure 3:**
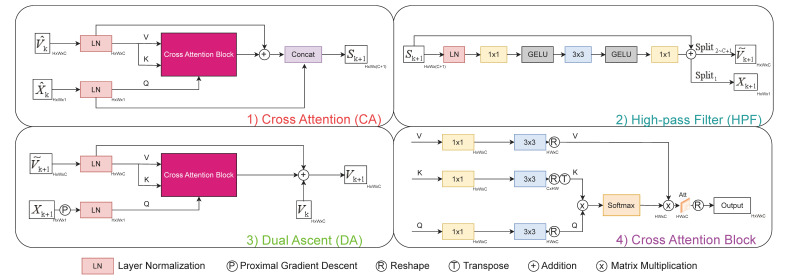
The architecture of Cross Attention (CA), Dual Ascent (DA), High-pass Filter (HPF) module and Cross Attention Block.

In the following sections, we will introduce the specific implementation of each module separately. Architectures of the modules are shown in Figure 3.

#### 3.2.2. Sampling and Initial Reconstruction

Traditional methods typically use fixed-window size Gaussian matrices as sampling modules, which have drawbacks such as lower sampling quality and the generation of artifacts during the sampling process. To address these limitations, we adopt the Cross-Block Strategy (CBS), as proposed in previous research [14,15,20], as our sampling module. In the CBS-based sampling process, we replace the manually designed matrix multiplication with a learnable convolutional layer, denoted as A(•). Specifically, for an image $X\in R^{H\times W}$, when the selected compressed ratio is $\frac{M}{N}$, the sampling process can be expressed as

(28) $$\begin{array}{c} Y=A\left(X\right)=W_{A}*X, \end{array}$$

where * denotes the bias-free and non-overlapping convolution operation. The convolution kernel $W_{A}$ is reshaped by the learnable sampling matrix $A\in R^{M\times N}$ into *M* filters, each of which is of kernel size $1\times \sqrt{N}\times \sqrt{N}$. The data measurement $Y\in R^{M\times \frac{H}{\sqrt{N}}\times \frac{W}{\sqrt{N}}}$ is then obtained.

For the initialization of the primal variable, we use the transpose operation $X_{0}=A^{T}\left(Y\right)$, implemented by a convolutional layer followed by a PixelShuffle layer, and extend this process to the entire image. This is defined as

(29) $$\begin{array}{c} X_{0}=A^{T}\left(Y\right)=\mathrm{Pixelshuffle}\left(W_{A^{T}}*Y\right). \end{array}$$

Here, $W_{A^{T}}$ is obtained by reshaping $A^{T}\in R^{N\times M}$ into *N* filters, each with a kernel size of M×1×1. The convolution operation $W_{A^{T}}*Y$ results in a tensor of size ${\left(\sqrt{N}\right)}^{2}\times \frac{H}{\sqrt{N}}\times \frac{W}{\sqrt{N}}$, after which the PixelShuffle layer reshapes this tensor into the desired H×W dimension.

Following this, two convolutional layers, each with a kernel size of 3×3 and *C* channels, are applied to $X_{0}$ to generate the initial dual variables $V_{-1},V_{0}\in R^{H\times W\times C}$, as denoted by

(30) $$\begin{array}{ccc} & \; & V_{-1}={\mathrm{Conv}}_{\Phi_{-1}}\left(X_{-1}\right), \end{array}$$

(31) $$\begin{array}{c} \begin{array}{cc} \; & V_{0}={\mathrm{Conv}}_{\Phi_{0}}\left(X_{0}\right). \end{array} \end{array}$$

#### 3.2.3. Cross Attention

To effectively integrate the information from the primal and dual spaces, we designed a Cross Attention (CA) module which is composed of two types of asymmetrical inputs. Since the primal and dual variables have different dimensions, this module addresses the need for dimensional alignment. As illustrated in the upper-left panel of Figure 3, inputs to the CA module are generated as follows:

The primal variable $X_{k}$ is first converted into variable ${\hat{X}}_{k}\in R^{H\times W}$ via a gradient descent operator, while the inertia term ${\hat{V}}_{k}\in R^{H\times W\times C}$ is obtained through an encoder for $V_{k}-V_{k-1}$ which consists of a convolutional layer with kernel size of 3×3. These processes can be expressed as the following:

(32) $$\begin{array}{ccc} \; & & {\hat{X}}_{k}=X_{k}-\rho A^{T}\left(A\left(X_{k}\right)-Y\right), \end{array}$$

(33) $$\begin{array}{c} \begin{array}{cc} \; & {\hat{V}}_{k}={\mathrm{Conv}}_{e}\left(V_{k}-V_{k-1}\right). \end{array} \end{array}$$

Unlike the original Transformer model [24,25], which uses three inputs Q,K,V for contextual dependency modeling, our CA module simplifies this by having only two asymmetrical inputs, i.e., by assuming that K=V. The variable ${\hat{X}}_{k}$ is used as Q, and ${\hat{V}}_{k}$ serves as both K and V in the CA module to generate the synthesized variable $S_{k}\in R^{H\times W\times (C+1)}$, expressed as

(34) $$\begin{array}{cc} \; & S_{k+1}={\hat{X}}_{k}⊕{\hat{V}}_{k}=\mathrm{CA}\left({\hat{X}}_{k},{\hat{V}}_{k}\right). \end{array}$$

As shown in the lower-right panel of Figure 3, the implementation of the CA module is similar to that of the Octuf [14]. Firstly, the inputs ${\hat{X}}_{k}$, ${\hat{V}}_{k}$ are, respectively, embedded by a 1×1 convolutional layer ${\mathrm{Conv}}_{Q,K,V}^{1\times 1}\left(•\right)$, respectively, to obtain features with the size being H×W×C. Despite the different input channels, the output channels are made identical. Next, a 3×3 convolutional layer ${\mathrm{Conv}}_{Q,K,V}^{3\times 3}\left(•\right)$ is used to encode channel-wise spatial context. Finally, the outputs are reshaped into tokens $\left\{\hat{Q},\hat{K},\hat{V}\right\}\in R^{HW\times C}$ using the reshape operation R(•). This process is described as follows:

(35) $$\begin{array}{cc} \; & \hat{Q}=R\left({\mathrm{Conv}}_{Q}^{3\times 3}\left({\mathrm{Conv}}_{Q}^{1\times 1}\left(Q\right)\right)\right), \end{array}$$

(36) $$\begin{array}{c} \begin{array}{cc} \; & \hat{K}=R\left({\mathrm{Conv}}_{K}^{3\times 3}\left({\mathrm{Conv}}_{K}^{1\times 1}\left(K\right)\right)\right), \end{array} \end{array}$$

(37) $$\begin{array}{c} \begin{array}{cc} \; & \hat{V}=R\left({\mathrm{Conv}}_{V}^{3\times 3}\left({\mathrm{Conv}}_{V}^{1\times 1}\left(V\right)\right)\right). \end{array} \end{array}$$

Subsequently, the attention variable $\mathrm{Att}\in R^{HW\times C}$ is obtained through the attention mechanism:

(38) $$\begin{array}{c} \mathrm{Att}=\hat{V}\mathrm{Softmax}\left({\hat{K}}^{T}\hat{Q}\right). \end{array}$$

This result is reshaped into a feature of size $R^{H\times W\times C}$. Then, an 1×1 convolutional layer ${\mathrm{Conv}}_{A}\left(•\right)$ is applied for enhancing the feature extraction. In each iteration layer, the synthesized variable $S_{k}$ is obtained by concatenating $X_{k}$ and ${\mathrm{Att}}_{k}$. The overall process of the CA module can be expressed as

(39) $$\begin{array}{c} \mathrm{CA}\left({\hat{X}}_{k},{\hat{V}}_{k}\right)=\mathrm{Concat}\left({\hat{X}}_{k},{\mathrm{Conv}}_{A}\left(R\left({\mathrm{Att}}_{k}\right)\right)\right). \end{array}$$

#### 3.2.4. High-Pass Filter

After combining the inertial information from the primal and dual spaces through the Cross Attention module, it is necessary to perform a high-pass filter (denoising) operation on the synthesized variable. Some studies [21] suggest that performing a high-pass filtering operation in the high-dimensional unfolding space often yields better reconstruction results. Our High-pass Filter consists of three layers of alternating 1×1 and 3×3 convolutional layers combined with the GELU function, as shown in Figure 3. The synthesized variable is encoded into a T×C dimensional space, and then a sparse solution is obtained through the GELU function before being decoded back into the T×C dimensional space. This operation can be expressed as

(40) $$\begin{array}{c} {\hat{S}}_{k+1}=\mathrm{HPF}\left(S_{k+1}\right). \end{array}$$

In the iterative algorithm, the high-pass filter effect is typically achieved using a form like $F^{-1}S_{\gamma }\left(F\left(x\right)\right)$, where F represents a sparse operator. And there exists a connection that $S_{\gamma }\left(x\right)=\mathrm{sgn}\left(x\right)\mathrm{ReLU}\left(\left|x\right|-\gamma \right)$, where the ReLU function [32] plays a similar role as high-pass filters with the GELU function. Similarly, our HPF module consists of an in-transformation and an out-transformation, with the GELU function acting as the high-pass filter, as shown in Figure 3. Previous studies [11,14] have demonstrated that data-driven convolutional layers can automatically learn the transformations into and out of the sparse space. Consequently, our High-pass Filter module essentially functions as a proximal mapping step with learnable parameters.

#### 3.2.5. Dual Ascent

Before executing the Dual Ascent module, we need to reduce the dimensions of the synthesized variable ${\hat{S}}_{k+1}$ to update the primal variable $X_{k+1}$, which is then used as the final output $X^{*}$ after all iteration layers. In this paper, we adopt a method similar to that of Outuf [14], using the ${\mathrm{Split}}_{1}\left(•\right)$ operator to extract the first dimension of the synthesized variable as the output for the primal variable, as shown in the lower-left panel of Figure 3. Through training of the neural network, this method can adaptively yield reasonable results, while avoiding aliasing [33] caused by the down-sampling of convolutional layers.

In the Dual Ascent module, to save parameters and improve performance without introducing new convolutional neural network or Transformer modules, we employ a novel approach. Specifically, we extract the remaining *C* dimensions of the synthesized variable, denoted as ${\tilde{V}}_{k+1}={\mathrm{Split}}_{2∼C+1}\left(S_{k+1}\right)$ to be used as a fixed input to the K,V positions of the CA module. These dimensions, considered as a whole, form the parameters of an encoder to update the dual variable $V_{k+1}$ by utilizing the residual information between *Y* and the primal variable $X_{k+1}$ during the iteration, which can be expressed as follows:

(41) $$\begin{array}{c} \begin{array}{cc} V_{k+1} & =V_{k}+\left[•⊕{\tilde{V}}_{k+1}\right]\left(A^{T}\left(A\left(X_{k+1}\right)-Y\right)\right)\\ \; & =V_{k}+{\mathrm{Split}}_{2∼C+1}\left(\left(A^{T}\left(A\left(X_{k+1}\right)-Y\right)\right)⊕{\tilde{V}}_{k+1}\right). \end{array} \end{array}$$

This method is reasonable for the following reasons:

1. Since the dimensions of the primal and dual spaces differ, to integrate information from the primal space into the dual space, we require an encoder with a dimension-matching mechanism. The dimensions of ${\tilde{V}}_{k+1}\in R^{H\times W\times C}$ and $V_{k}\in R^{H\times W\times C}$ are always the same, so using ${\tilde{V}}_{k+1}$ as a fixed input naturally matches the dimensions of the primal variable with those of the dual space via the preceding CA module. Additionally, this approach leverages the information lost during down-sampling via the ${\mathrm{Split}}_{1}\left(•\right)$ operator, resulting in better performance than using fixed coefficients as the encoder;
2. We note that, in Equation (22), the dual-ascent term requires the fixed coefficients ρ and $\frac{\rho }{1+\rho }$ to be positive-definite. Our DA module employs an attention mechanism with identical K and V inputs. To ensure strict positive definiteness when the encoder operates on the dual-ascent term, one approach is to use a linear attention mechanism [34], which produces the linear attention variable ${\mathrm{Att}}_{L}$. The process is outlined as follows:
  Assume that for feature maps $\left\{X,V\right\}\in R^{hw\times c}$, it follows that $\left\{x_{j},v_{i}\right\}\in R^{hw\times 1}$, for (i,j∈1,2,⋯,c),
  (42) $$\begin{array}{c} \begin{array}{cc} {\mathrm{Att}}_{L}=V\left(V^{T}X\right) & =\left(v_{1},v_{2},\cdots ,v_{c}\right)\left(\begin{array}{cccc} v_{1}^{T}x_{1} & v_{1}^{T}x_{2} & \cdots & v_{1}^{T}x_{c}\\ \cdots & \cdots & \cdots & \cdots \\ v_{c}^{T}x_{1} & v_{c}^{T}x_{2} & \cdots & v_{c}^{T}x_{c} \end{array}\right)\\ \; & =\left(\begin{array}{cccc} \sum_{i=1}^{c}{∥v_{i}∥}^{2}x_{1} & \sum_{i=1}^{c}{∥v_{i}∥}^{2}x_{2} & \cdots & \sum_{i=1}^{c}{∥v_{i}∥}^{2}x_{c} \end{array}\right), \end{array} \end{array}$$
  The coefficient term $\sum_{i=1}^{c}{∥v_{i}∥}^{2}$ is clearly positive-definite. This is established based on prior research [34], which shows that the standard attention mechanism can achieve equal or even superior performance compared to the linear attention mechanism. We argue that the encoder derived from this approach can approximately maintain positive definiteness, thereby supporting the preservation of the dual ascent method’s acceleration characteristics in DAT.

#### 3.2.6. Loss Function

For an original image $X_{i}$ in the dataset, of which the batchsize is $N_{b}$, the model first obtains the CS measurement values $Y_{i}$ through sampling, then predicts the reconstructed image $X_{i}^{*}$. We perform end-to-end optimization of our DAT through the following loss function:

(43) $$\begin{array}{c} \ell_{\theta }\left(X_{i}^{*},X_{i}\right)=\ell_{{\mathrm{mse}}_{\theta }}\left(X_{i}^{*},X_{i}\right), \end{array}$$

where $\ell_{{\mathrm{mse}}_{\theta }}$ is the mean squared error (MSE) between the original image $X_{i}$ and the reconstructed image $X_{i}^{*}$. During the entire training process, we traverse over all the images in each batch, summing up their loss functions to obtain our total loss $\left(i\in 1,2,\cdots ,N_{b}\right)$:

(44) $$\begin{array}{c} \ell_{{\mathrm{total}}_{\theta }}=\sum_{i=1}^{N_{b}}\ell_{\theta }\left(X_{i}^{*},X_{i}\right), \end{array}$$

which is the objective function we aim to optimize.

## 4. Experimental Results

### 4.1. Details of Implementation

For training, we used 400 images from the training and test datasets of the BSD500 dataset [35]. The training images were cropped into 89,600 patches of size 96 × 96 pixels and augmented following those in the previous study [36]. In the sampling module, the blocksize $\sqrt{N}$ is set to 32, i.e., N=1024. For each given CS ratio $\frac{M}{N}$, respectively, 0.1, 0.3, and 0.5, a corresponding learnable measurement matrix *A* is constructed by using a convolutional layer with a kernel size of $M\times 1\times \sqrt{N}\times \sqrt{N}$ to sample the original image $X\in R^{96\times 96}$ to obtain the measurement $Y\in R^{M\times 9}$. Then, we apply the transpose of the sampling matrix as the kernel weights for convolution to *Y* to obtain the the initial reconstruction $X_{0}$.

For the network parameters, the default dimension of dual variable *C* is 31, the dimension expansion factor *T* is 4, the number of iteration layers *K* is 10, and the batch size is 8. The size of the unspecified convolution kernel is 3×3. We use the Adam optimizer [37] to train the network with the initial learning rate, which is decreased to $5\times 10^{-5}$ through 100 epochs using the cosine annealing strategy [38] and the warm-up epochs are 3. For testing, we utilize a widely used benchmark dataset, yielding Set11 [9]. Two common-used image assessment criteria, Peak Signal to Noise Ratio (PSNR) and Structural Similarity (SSIM) [39], are used to evaluate the reconstruction results.

All the experiments are implemented in PyTorch 2.0.1+cu118 with an RTX 3090 GPU (NVIDIA Corporation, Santa Clara, CA, USA), except for the reconstruction time analysis, which was conducted using an RTX 4080 Laptop GPU from the same manufacturer.

### 4.2. Early-Stage Performance

We compared our model with other advanced models, particularly those utilizing the Transformer framework and dimension expansion strategy, namely Octuf [14], TransCS [15], and CSformer64 [16]. To evaluate the early-stage performance of the models, we examined the loss function values at each epoch, the actual reconstruction quality, and the number of parameters. For the competing methods, we migrated their models from the source code into our program, ensuring they operated within the same training–testing environment. We then retrained them and recorded the results for the first 10 epochs.

The selection of the initial learning rate closely follows the settings from the original papers. However, to enable the competing methods to achieve better and more reliable performance, we made the following adjustments: the initial learning rate for Octuf is set to $4.8\times 10^{-4}$, for TransCS it is $1.0\times 10^{-4}$, for CSformer64 it is $2.0\times 10^{-4}$, and for our model, DAT, it is $4.8\times 10^{-4}$.

#### 4.2.1. Training Process Analysis

**Figure 4:**
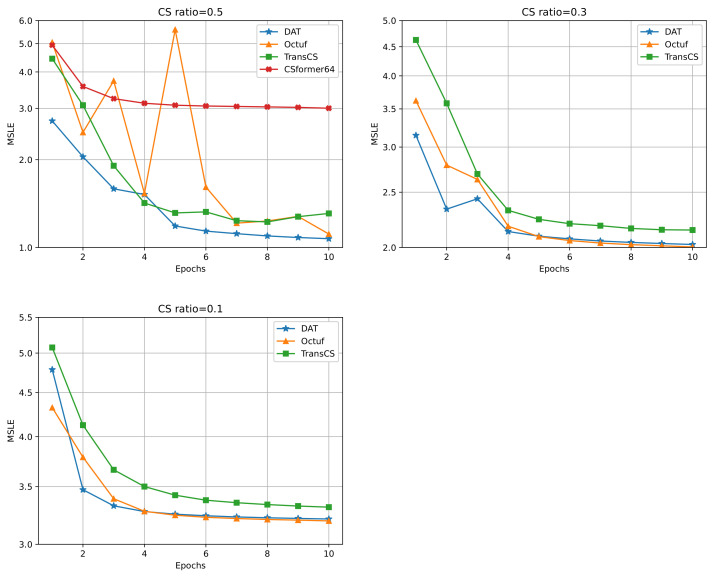
Comparison of training Mean Squared Logarithmic Error (MSLE) in first 10 epochs in the case of CS ratios = 0.5, 0.3, and 0.1 respectively.

In Figure 4, we compare the training processes of DAT with competing methods under CS ratios of 0.5, 0.3, and 0.1, using Mean Squared Logarithmic Error (MSLE) as the evaluation metric, which is calculated as $\ln(\ell_{{\mathrm{total}}_{\theta }})$. (For CSformer, the original authors provided source code only for a CS ratio of 0.5.) We observed that, for most epochs, our model’s curve remained at the lowest position. These results indicate that our model achieves a good initial MSLE and reaches the smallest training MSLE, i.e., the best performance during early-stage training. Specifically, during the first four epochs, at a CS ratio of 0.5, when comparing the average MSLE values of the models, our model (1.96) outperformed the competing methods, which had MSLE values of 3.19, 2.71, and 3.71, respectively, achieving reductions of 1.23, 0.75, and 1.75.

#### 4.2.2. Comparison of Reconstructions

Although DAT’s MSLE may exceed that of Octuf during epoch 1 and the final training stages at CS ratios of 0.1 and 0.3, we also employ another widely used metric, PSNR, to evaluate the actual reconstruction quality of the models. As shown in Table 1, we assessed the average PSNR of reconstructed images on Set11 for all models at epochs 1 and 10. For instance, under a CS ratio of 0.1 at epoch 1, although Figure 4 shows that our training MSLE is higher than that of Octuf, our model successfully outperformed competing methods by 1.64 dB (6.04%) and 5.53 dB (20.37%), respectively.

**Figure 5:**
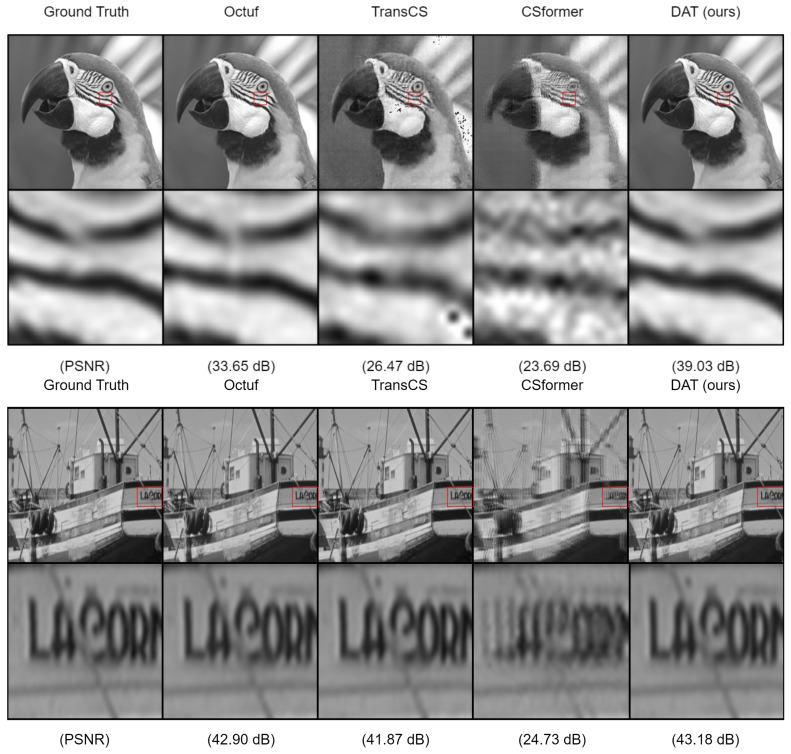
Comparison of image reconstruction from the Set11 dataset by the Epoch-1 Models (**Top**) and Epoch-10 Models (**Bottom**) in the Case of CS Ratio = 0.5.

Overall, in terms of the early-stage training results that we focus on, specifically at epoch 1, our model achieved higher average PSNR across all three CS ratios compared to the competing methods, with improvements of 3.53 dB (10.59%), 9.27 dB (27.81%), and 10.66 dB (31.98%). This observation aligns with the trends we noted in MSLE and further validates that, even when DAT occasionally exhibits higher MSLE than competing methods, the images reconstructed by our model still achieve the best quality. This highlights the excellent generalization ability of our model. Figure 5 presents the visual comparisons on challenging images.

A similar trend can be observed in the PSNR evaluations at epoch 10. Although under a CS ratio of 0.1, DAT’s MSLE and PSNR were slightly inferior to those of Octuf, our model still delivered the best results in terms of average reconstructed PSNR, demonstrating its overall superiority.

#### 4.2.3. Comparison of Convergences and Time Complexities

To define convergence criteria more concretely, we used the PSNR of ISTA-Net+ [11] on the Set11 dataset as a benchmark. As shown in Table 2, we evaluated the number of epochs required for each model to first exceed ISTA-Net+’s performance. At CS ratios of 0.5, 0.3, and 0.1: DAT required only one epoch at most, Octuf required up to four epochs, TransCS required up to five epochs, and CSformer required more than ten epochs.

We acknowledge that comparing the training speed of different models also necessitates considering the training time per epoch. Notably, for all models, training time is always proportional to reconstruction time. Therefore, we use the average reconstruction time for 256 × 256 images on the Set11 dataset as a proxy for training time to analyze time complexity, as shown in the first column on the right side of Table 2.

Although our model exhibits a disadvantage in this metric, its reconstruction speed remains within the same order of magnitude as state-of-the-art lightweight models, making this an acceptable trade-off. To provide a more objective comparison of training speed—our primary focus—we introduce the Time-Epoch Product (TEP) metric, calculated as runtime × epoch. As shown in the second column on the right side of Table 2, this metric clearly demonstrates that our model achieves the fastest training speed under the given convergence criteria, with a TEP (absolute time consumption) at most one-third that of competing models.

#### 4.2.4. Comparison of Model Sizes

Finally, we conducted a model size comparison, taking the compressed sensing ratio of 0.5 as a example. As shown in the rightmost column of Table 2, even though the competing methods in this comparison are all advanced models with relatively few parameters, ranging from 0.82 M to 2.28 M, our model achieves the aforementioned performance while having the fewest parameters, at only 0.76 M. Fewer parameters imply that our model also has the lowest training complexity, which reflects the success of our parameter reuse strategy.

### 4.3. Influence of Initial Learning Rate

The initial learning rate is one of the critical parameters that determine the performance of deep neural network models. However, as a hyperparameter, there is often no robust theoretical basis for selecting an appropriate initial learning rate. If a neural network model with the same structure exhibits significant performance variations due to the choice of the initial learning rate, we consider the model’s performance to lack robustness with respect to this parameter. For user-specific customization needs, a truly excellent model should exhibit strong robustness to changes in the initial learning rate, ensuring reliable performance across a range of learning rate settings.

To investigate this, we compared DAT with Octuf, a model with similar performance to DAT in previous experiments. As shown in Table 3, under the CS ratio of 0.5, we set the initial learning rates for both models within the range of $4.0\times 10^{-4}$ to $5.0\times 10^{-4}$, with an interval of $0.2\times 10^{-4}$, and trained each model separately. Three metrics were used for evaluation: maximum MSE, minimum MSE, and final MSE. Maximum MSE reflects the worst fluctuations observed during training. A large value indicates poor convergence stability under the current initial learning rate, potentially leading to significantly longer training epochs. Minimum MSE indicates the best performance achievable by the model with the given initial learning rate. A smaller value suggests that the model can converge to a good extremum point. The difference between the maximum and minimum MSE reflects whether there are fluctuations during the learning process. A smaller difference indicates fewer fluctuations, suggesting a more stable learning process. Final MSE also reflects learning stability. If this value exceeds the minimum MSE, it suggests that the model experienced fluctuations, and the previously reached extremum was a narrow optimum rather than a wide optimum, which is detrimental to convergence.

To quantify the impact of variations in the initial learning rate on the aforementioned three metrics, we calculated their mean and variance. As shown in Table 3, the average difference between the maximum and minimum MSE for the competing method Octuf is 1697.3, and its average final MSE exceeds the minimum MSE. In contrast, DAT exhibits an average difference of only 30.63 between the maximum and minimum MSE, and its average final MSE is nearly identical to the minimum MSE. This indicates that our model maintains a more stable and superior training process regardless of the initial learning rate. Moreover, we compared the variance of the three metrics mentioned above, finding that the variances for the competing methods are 4878, 209, and 220 times larger than those of our model, respectively. A larger variance indicates that the training stability of the model varies significantly with changes in the initial learning rate. In contrast, the variances for our model are much smaller, demonstrating that our model not only maintains an overall more stable training process but also exhibits less sensitivity to changes in the initial learning rate.

The stability of model training ultimately impacts the effectiveness of early-stage training. Taken together, these results show that our model’s performance is highly robust to the selection of the initial learning rate and significantly outperforms the competing method in this regard, making it much more user-friendly for practical applications.

## 5. Conclusions

In this paper, we propose a novel Dual-Ascent-Inspired Transformer (DAT) for Compressed Sensing. Specifically, we replace the addition of the inertial term in AADMM with a Cross-Attention module and reuse the parameters of this module during the dual ascent steps. This approach reduces training complexity while preserving the key characteristics of the dual-ascent method that accelerate convergence. This is achieved through the excellent encoding properties of the Cross-Attention module and the approximate positive definiteness demonstrated by the Dual Ascent module. Experiments show that our DAT can achieve comparable results with less training data compared to state-of-the-art methods, and the training process is more stable and less sensitive to the selection of the initial learning rate, making it more suitable for the needs of individual users to customize models. In the future, we will explore the relationship between initial convergence and long-term training performance. Indeed, preliminary evidence suggests a positive correlation between early convergence and the model’s long-term performance, indicating that better deep compressed sensing models can be developed based on the DAT framework. We plan to incorporate novel sampling modules and leverage the strengths of other reconstruction algorithms, aiming for breakthroughs in classic reconstruction metrics.

## Figures and Tables

**Table 1:** Average PSNR (dB) and SSIM performance comparisons on the Set11 dataset [9] at the first and tenth epochs under different CS ratios. The best results are highlighted in red and our model’s results are highlighted in blue.

	Epoch 1				Epoch 10			
Method	CS Ratio							
	10%	30%	50%	Avg	10%	30%	50%	Avg
Octuf	25.51/0.7983	30.45/0.8842	33.44/0.9525	29.80/0.8783	30.18/0.8977	36.76/0.9650	40.76/0.9826	35.90/0.9485
TransCS	21.62/0.5815	24.33/0.7071	26.24/0.7752	24.06/0.6879	29.03/0.8776	35.71/0.9587	39.85/0.9794	34.86/0.9386
CSformer	-	-	22.67/0.6529	22.67/0.6529	-	-	24.22/0.7590	24.22/0.7590
DAT(Ours)	27.15/0.8351	33.93/0.9515	38.82/0.9782	33.33/0.9216	29.99/0.8928	36.77/0.9651	40.95/0.9830	35.90/ 0.9469

**Table 2:** Comparison of convergence speed, computational efficiency, and model size across different CS ratios on the Set11 dataset. The left part presents the number of epochs and corresponding PSNR (dB) required to reach ISTA-Net+ performance under different CS ratios, while the right part provides runtime, TEP, and parameter count under a CS ratio of 0.5. The best results are highlighted in red.

Method	CS Ratio				Run Time	TEP	Param
	10%	30%	50%				
Octuf	2/27.72	3/34.79	4/38.63		0.046s	0.184	0.82M
TransCS	4/27.63	4/34.49	5/38.44		0.039s	0.195	2.28M
CSformer	-	-	10+		0.021s	0.210+	1.76M
DAT(Ours)	1/27.15	1/33.93	1/38.82		0.060s	0.060	0.76M
ISTA-Net+	20/26.64	20/33.82	20/38.07		0.016s	0.320	1.70M

**Table 3:** Comparison of maximum, minimum, and final MSE during training in the case of CS ratio = 0.5 across different initial learning rates, along with their averages and variances. The best results are highlighted in red.

Method	Indicator	Initial Learning Rate							
		$4.0\times 10^{-4}$	$4.2\times 10^{-4}$	$4.4\times 10^{-4}$	$4.6\times 10^{-4}$	$4.8\times 10^{-4}$	$5.0\times 10^{-4}$	Avg	Var
Octuf	MaxMSE	64.82	3663.50	309.35	827.26	267.35	5077.60	1701.65	4,537,075.99
	MinMSE	2.90	3.03	2.86	6.90	3.35	7.06	4.35	4.18
	FinalMSE	2.96	3.03	2.86	9.60	3.58	7.86	4.98	8.80
DAT(Ours)	MaxMSE	24.71	20.33	20.90	95.44	15.12	25.30	33.63	930.20
	MinMSE	2.94	2.95	3.25	2.89	2.91	3.07	3.00	0.02
	FinalMSE	2.94	2.95	3.42	2.89	2.91	3.07	3.03	0.04

## Data Availability

Data are contained within the article.
